# Acute on Chronic Theophylline Toxicity in an Elderly Patient

**DOI:** 10.7759/cureus.13484

**Published:** 2021-02-22

**Authors:** Alexander Kong, Somshukla Ghosh, Chelsea Guan, Brittany L Fries, Floyd W Burke

**Affiliations:** 1 Internal Medicine Residency, University of Central Florida-HCA Healthcare Graduate Medical Education (GME), Orlando, USA; 2 Internal Medicine, University of Central Florida College of Medicine, Orlando, USA; 3 Clinical Pharmacy, Orlando Veterans Affairs (VA) Medical Center, Orlando, USA; 4 Cardiology, Orlando Veterans Affairs (VA) Medical Center, Orlando, USA

**Keywords:** theophylline, toxicity, wide-complex tachycardia

## Abstract

Theophylline toxicity has become rare in the 21st century, as the drug has fallen out of favor due to serious life-threatening adverse events, narrow therapeutic window and readily available therapeutic alternatives. The wide array of clinical symptoms related to theophylline toxicity makes this diagnosis challenging for the treating physician. We report a case of an elderly gentleman who presented with respiratory failure and seizures due to severe theophylline toxicity.

## Introduction

Theophylline is a methylxanthine used for the treatment of respiratory diseases, such as asthma, chronic obstructive pulmonary disease (COPD) and apnea of prematurity, due to its pharmacological action in smooth muscle relaxation, anti-inflammatory properties and powerful stimulation of the central nervous system (CNS) respiratory center [1]. The narrow therapeutic range (serum concentration 10 to 20 mcg/mL for adults) and the concomitant use of certain medications that impair theophylline clearance make individualized dosing critical and underscore the need for frequent serum theophylline concentration monitoring [2-4]. Life-threatening toxicity is associated with serum concentrations of 80 to 100 mcg/mL in acute overdoses and 40 to 60 mcg/mL in chronic overdoses [5]. Additionally, acute-on-chronic toxicity can occur with acute overdoses in adults already being treated with therapeutic theophylline [6]. Life-threatening theophylline toxicity remains a concern throughout treatment duration, especially in the elderly due to potential alterations in theophylline pharmacokinetics, increase in interacting medications and/or improper adherence resulting in overdosing [4,6,7].

Theophylline toxicity can present with a wide array of clinical manifestations, ranging from mild gastrointestinal complaints to potentially lethal cardiac arrhythmias and seizures. Its non-specific symptom presentations combined with the decreased use of theophylline in the 21st century make it a diagnostic challenge, requiring a high level of suspicion [8]. This is true even though the pharmacokinetics, clinical syndrome and risk factors for developing theophylline toxicity are well documented in the literature.

We present a case of an elderly patient, treated chronically with theophylline for severe asthma-COPD overlap, who developed severe theophylline toxicity in the setting of an acute exacerbation. The aim of this report is to provide a current, problem-based example of the typical clinical picture of theophylline toxicity in order to increase physician awareness of this infrequent entity, reinforce the importance of patient education and routine drug level monitoring in long-term therapy and in acute exacerbations.

## Case presentation

**Figure 1:**
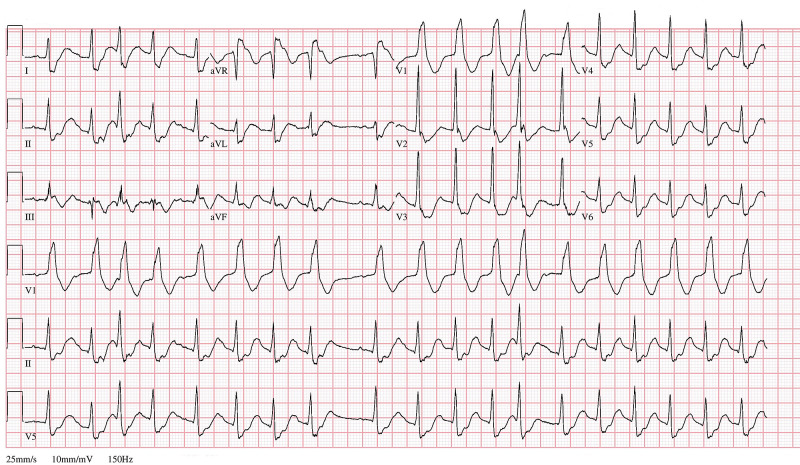
Electrocardiogram showing wide-QRS-complex tachycardia and right bundle branch pattern.

An 83-year-old male with a past medical history of coronary artery disease, hypertension, severe persistent asthma, COPD GOLD stage 2D, gastroesophageal reflux disease and hypothyroidism presented to the emergency department due to an episode of witnessed generalized tonic-clonic seizure that was preceded by a one-day history of non-bloody, non-bilious vomiting and throat tightness. On presentation, the patient was agitated, oriented to only himself and showed significantly increased work of breathing with the use of accessory muscles. Vital signs showed a temperature of 97.4 °F, blood pressure of 134/81 mmHg, heart rate of 146 beats per minute and respiratory rate of 26 breaths per minute. Mucous membranes were dry, and he had decreased skin turgor. The respiratory exam showed good chest excursions, poor air entry at the bases with minimal end-expiratory wheezing at the left upper lung field. The cardiovascular exam was unremarkable other than sinus tachycardia. Neurologically, the patient was alert, awake, oriented with normal speech and mentation, moving all extremities with preserved motor strength. Electrocardiogram (Figure 1) and telemetry showed wide-QRS-complex tachycardia that was unresponsive to intravenous adenosine. A basic metabolic panel (Table 1) showed combined metabolic and respiratory acidosis, hypokalemia with a potassium level of 2.2 mEq/L, acute kidney injury with a creatinine of 1.4 mg/dL (baseline 0.8 mg/dL) and thyroid-stimulating hormone level of 0.829 mIU/L.

**Table 1:** Basic metabolic panel on initial presentation. Local lab reference range in parenthesis. EGFR: estimated glomerular filtration rate.

Basic metabolic panel	
Glucose	267 mg/dL (70-105)
Urea nitrogen	24 mg/dL (7-20.6)
Creatinine	1.4 mg/dL (0.7-1.3)
Sodium	134 mEq/L (136-145)
Potassium	2.2 mEq/L (3.5-5.2)
Chloride	98 mEq/L (98-109)
CO_2_	18 mmol/L (22-31)
Calcium	10 mg/dL (8.4-10.6)
EGFR	48 mL/min (>60)

The patient was admitted to the intensive care unit for monitoring. On serial examination, the patient developed seizure activity, becoming non-responsive, unable to protect his airway and required emergent rapid-sequence intubation and mechanical ventilation.

The patient’s medication reconciliation (see Appendix, Table 2) included extended-release theophylline 300 mg capsules once daily, which he had been taking as usual (last administration day of presentation). There were no new medication changes with the exception of a recent course of sulfamethoxazole/trimethoprim tablets for a urinary tract infection. On laboratory testing, theophylline level was supratherapeutic at 80.7 mcg/mL (therapeutic range 10-20 mcg/mL). The patient was treated for theophylline toxicity with hemodialysis without ultrafiltration, for a total of seven hours. Serum theophylline level gradually lowered to therapeutic levels, with the resolution of electrolyte abnormalities, metabolic acidosis and cardiac arrhythmias. The patient did not have any further episodes of seizures. Mental status progressively improved and he was able to be extubated on hospital day 4. A severe adverse reaction to theophylline was documented in the patient’s electronic medical record and theophylline was permanently discontinued.

## Discussion

Given high mortality risk if cardiac arrhythmias or seizures are present, suspicion for theophylline toxicity at entry into the Emergency Department, and prompt initiation of emergency management is paramount for favorable outcomes, particularly in the elderly [4,6]. Physicians must have a high index of suspicion and medication reconciliation is of the utmost importance [9]. In our patient, sub-optimal communication with the patient’s spouse and spouse misunderstanding of medication therapy delayed historical evidence of theophylline use.

Our patient presented with a constellation of gastrointestinal, cardiovascular, central nervous systems and metabolic derangements consistent with hyperadrenergic stimulation [10]. Toxic theophylline levels cause phosphodiesterase inhibition and consequently increase systemic levels of cyclic adenosine monophosphate, which augments beta-adrenergic effects. Its combined effect as an adenosine antagonist promotes smooth muscle relaxation, myocardial stimulation, increased chronotropism and inotropism, CNS excitation, increased muscle oxygen consumption and increased basal metabolic rate eventually leading to metabolic acidosis [5].

The management of this patient was initially symptom-based. Cardiac monitoring showed wide QRS complex tachycardia that was not responsive to intravenous adenosine, however at that time theophylline toxicity was not suspected. Adenosine has been recommended as first-line therapy by some experts to reverse theophylline-induced supraventricular tachycardia (SVT). However, given theophylline acts as an adenosine antagonist, failure to convert a methylxanthine-induced SVT with adenosine is common [5]. In addition, adenosine should be used with extreme caution in patients with asthma or COPD, given it poses a paradoxical risk for bronchoconstriction. Even though SVT and other atrial tachycardias are the most common arrhythmias induced by theophylline, the patient’s severe electrolyte disturbances could have also caused ventricular tachycardia [6]. The electrolyte disturbances were likely secondary to both surreptitious vomiting and theophylline toxicity.

Our patient’s respiratory depression could have been due to transient seizures or directly related to theophylline toxicity. Severe theophylline toxicity can cause hyperventilation, respiratory alkalosis, respiratory failure, respiratory arrest and acute respiratory distress syndrome [5]. Nonetheless, severe transient respiratory changes with hypercapnia and hypoxemia can also be present during seizure episodes [11]. In our case, our patient presented with tachypnea, likely related to direct ventilatory overstimulation by theophylline, and later seizure-induced respiratory depression leading to acute hypercapnic respiratory failure.

There are no antidotes to reverse the toxic effects of theophylline; therefore, therapy is focused on enhancing its elimination. In acute overdose, gastric decontamination with multi-dose activated charcoal (MDAC) is initially recommended if the patient is able to protect the airway, although it is often limited by intractable vomiting or ineffectiveness at reducing theophylline concentrations [12]. Extracorporeal treatment (ECTR) is more effective at removing theophylline than MDAC and hemodialysis is preferred over hemoperfusion [3,12,13]. ECTR is indicated at theophylline concentrations over 100 mcg/mL in acute exposure and suggested for theophylline concentration over 60 mcg/mL in chronic exposure. A lower threshold (over 50 mcg/mL) has been suggested if the patient is less than six months or more than 60 years old. ECTR is also indicated based on symptoms such as life-threatening dysrhythmias, seizures, mental status changes or hypotension [12]. Some recommendations are based on contraindications to administer MDAC, serious comorbidities or coexisting conditions such as COPD, end-stage renal disease and liver failure [12]. Our patient fulfilled multiple criteria for ECTR due to altered mental status, seizure activity, electrolyte abnormalities, wide-complex tachycardia, theophylline concentration of 80.7 mcg/mL, elderly age, numerous comorbidities and suspected acute-on-chronic toxicity. The patient underwent hemodialysis without ultrafiltration for seven hours during which he rapidly reverted to sinus rhythm, his serum electrolytes were corrected, and his seizures resolved.

This patient’s supratherapeutic theophylline level was attributed to self-administered inadvertent overuse of his chronic therapy triggered by an acute exacerbation of his asthma-COPD overlap. Hyperthyroidism may increase metabolism of theophylline and likewise, metabolism may be reduced with hypothyroidism. Thyroid replacement may affect theophylline clearance in patients that are not stabilized (i.e. euthyroid); however, our patient's most recent TSH was 0.829 mIU/L and, therefore, this was not suspected to have been a major contributing factor. Ipriflavone, a component of his antioxidant blend, has been associated with increased theophylline levels in two cases, thought to be due to a decrease in CYP 1A2 metabolism. Similarly, the black pepper component of turmeric compounds may be associated with increased absorption and reduced elimination of theophylline; however, these mechanisms are theoretical and poorly documented [14]. Recent sulfamethoxazole/trimethoprim and concurrent non-steroidal anti-inflammatory agents may have contributed to acute kidney injury, which may have resulted in reduced theophylline clearance. Since theophylline undergoes extensive hepatic metabolism, mediated by cytochrome P450 enzymes (CYPs), it can be enhanced by CYP inducers, such as phenytoin or phenobarbital, or reduced by CYP inhibitors, such as cimetidine and macrolides [15]. These CYPs have also been suggested to become inhibited in the setting of upper respiratory disease with associated fever [16]. The American Geriatric Society Beers Criteria warns that the concurrent use of cimetidine or ciprofloxacin with theophylline increases the risk of theophylline toxicity [17]. Over-the-counter (OTC) vitamin and herbal supplement use could have potentially interfered with theophylline metabolism. It is important to note that interactions between dietary supplements and prescription drugs are usually unknown as most of these supplements are not regulated by the US Food and Drug Administration [18]. Healthcare providers should remain vigilant and educate patients on the potential risks of OTC pharmaceuticals or “nutraceuticals”, especially when taking drugs with a narrow therapeutic index.

In our case, patient education on theophylline use and increased awareness of chronic theophylline toxicity may have helped prevent unintentional toxicity. Drug level monitoring is strongly advised at initiation, dosing changes, routinely (at least yearly) and when other medications are added, changed or discontinued, or there is a new illness that may alter theophylline clearance [2]. Our patient did not have theophylline serum monitoring in over three years despite continued refills and close follow up. In general, we recommend that prescription for this medication should always be individualized and reconsidered, given the high risk for life-threatening adverse effects and multiple, safer therapeutic alternatives for bronchodilation in the market.

## Conclusions

Theophylline toxicity is a life-threatening, challenging diagnosis due to its wide array of multi-organ non-specific clinical symptoms. Changes in mental status and lack of family availability may further hinder the diagnosis if an updated medication list is unavailable. A high index of suspicion is necessary for the prompt implementation of emergent procedures to reduce morbidity and mortality. Due to its narrow therapeutic index, prescribing theophylline should be strongly reconsidered, and if prescribed, the risk and benefits should be thoroughly discussed with the patient.

## Appendices

**Table 2:** Medications and supplements retrieved from the patient’s home. PO: orally, INH: oral inhalation, QD: daily, BID: twice daily, TID: three times daily, QID: four times daily, QHS: at bedtime, PRN: as needed, tab: tablet, cap: capsule, SA: sustained action.

Medications and supplements retrieved from the patient’s home.
1 empty bottle Rx sulfamethoxazole 800 mg-trimethoprim 160 mg 1 tab PO QD
1 unlabeled bottle Rx with unidentified pills
Amlodipine besylate 10 mg 1 tab PO QD
Aspirin 81 mg 1 tab PO QD
Blue Green Algae (AFA) Aphanizomenon flos-aquae Powder 1 cap PO QD
Cow colostrum/chicken egg yolk/zinc/mushroom/inositol/phytosterol blend 1 cap PO QD
Docusate sodium 100 mg 1 cap PO BID
Ferrous gluconate 324 mg 1 tab PO TID
Hydroxyzine HCl 10 mg 2 tab PO QHS
Melatonin 10 mg 1 tab PO QHS
Melatonin/fruit/dogwood/zinc/selenium sleep aid 1 tab PO QHS
Methocarbamol 500 mg 1 tab PO QID
Mometasone furoate inhalation powder 220 mcg 2 puffs INH BID
Multivitamin/coenzymeQ10/Fatty acid/mushroom/antioxidant blend 1 cap PO QD
Naproxen 500 mg 1 tab PO BID
Omeprazole 20 mg 2 cap PO BID
Oxymetazoline HCl 0.05% nasal spray 1 application in each nostril PRN BID
Potassium chloride 20 MEq SA 1 tab PO QD
Simethicone 80 mg 1 tab PO TID
Sucralfate 1 g tab PO QID
Theophylline 300 mg SA 1 cap PO QD
Turmeric powder 500 mg 1 cap PO BID
